# German, Austrian, and Swiss guidelines for systemic treatment of gastric cancer

**DOI:** 10.1007/s10120-023-01424-y

**Published:** 2023-10-17

**Authors:** Florian Lordick, Salah-Eddin Al-Batran, Dirk Arnold, Markus Borner, Christiane J. Bruns, Wolfgang Eisterer, Gerhard Faber, Ines Gockel, Dieter Köberle, Sylvie Lorenzen, Markus Möhler, Ron Pritzkuleit, Michael Stahl, Peter Thuss-Patience, Ewald Wöll, Thomas Zander, Georg Maschmeyer, For the Germany Society of Hematology and Medical Oncology (DGHO, Austrian Society of Hematology and Oncology (OeGHO), Swiss Society of Hematology (SSH), Swiss Society of Medical Oncology (SGMO); in collaboration with the Arbeitsgemeinschaft Internistische Onkologie (AIO) of the German Cancer Society

**Affiliations:** 1 Department of Medicine II (Oncology, Gastroenterology, Hepatology, and Pulmonology), University of Leipzig Medical Center, Liebigstr. 22, 04103 Leipzig, Germany; 2 Frankfurt, Institut Für Klinisch-Onkologische Forschung (IKF), UCT-Universitäres Centrum Für Tumorerkrankungen, Frankfurt, Germany; 3 Asklepios Tumorzentrum Hamburg, Asklepios Klinik Altona, Hamburg, Germany; 4 ONCOCARE Am Engeriedspital, Berne, Switzerland; 5 Universitätsklinikum Köln, Cologne, Germany; 6 Allgemein Öffentliches Klinikum, Klagenfurt am Wörthersee, Klagenfurt, Austria; 7 Celenus Teufelsbad Fachklinik, Blankenburg, Germany; 8 St.Claraspital, Basel, Switzerland; 9 Klinikum Rechts der Isar, TU München, Munich, Germany; 10 University Hospital Mainz, Mainz, Germany; 11 Cancer Registry Schleswig-Holstein, Kiel, Germany; 12 Evang. Huyssens-Stiftung Kliniken Essen-Mitte, Essen, Germany; 13 Vivantes Klinikum Berlin-Friedrichshain, Berlin, Germany; 14 St. Vinzenz-Klinikum, Zams, Austria; 15 Deutsche Gesellschaft Für Hämatologie und Medizinische Onkologie, Berlin, Germany

**Keywords:** Gastric cancer, Perioperative, Neoadjuvant, Chemotherapy, Immunotherapy, Targeted therapy

## Abstract

The updated edition of the German, Austrian and Swiss Guidelines for Systemic Treatment of Gastric Cancer was completed in August 2023, incorporating new evidence that emerged after publication of the previous edition. It consists of a text-based “Diagnosis” part and a “Therapy” part including recommendations and treatment algorithms. The treatment part includes a comprehensive description regarding perioperative and palliative systemic therapy for gastric cancer and summarizes recommended standard of care for surgery and endoscopic resection. The guidelines are based on a literature search and evaluation by a multidisciplinary panel of experts nominated by the hematology and oncology scientific societies of the three involved countries.

## Preface

Outcomes of patients with cancer depend highly on access to high-quality care. Part of the established quality-of-care criteria is adherence to evidence-based treatment recommendations. To provide practising oncologists in the three German-speaking countries in Europe, comprising a population of approximately 100 million inhabitants, with up-to-date evidence-based guidelines for patient care, the scientific German, Austrian, and Swiss societies of hematology and oncology nominated a multidisciplinary group of experts to revise consensus-based oncology treatment guidelines based on available scientific evidence. This process is coordinated by the German Society of Hematology and Medical Oncology (DGHO). Here, we report on the treatment recommendations from the latest version of the multidisciplinary guidelines for gastric cancer (Onkopedia), finalized in August 2023. This article focusses on locally advanced and metastatic stages (IB-IV). In summary, systemic perioperative chemotherapy is recommended as a mainstay of treatment for patients presenting with localized gastric cancer (stages IB-III). In stage IV gastric cancer patients, treatment goals are palliative in most patients. Sequential lines of chemotherapy have shown to provide the best chances for prolonging patients’ survival, providing symptom control and lead to a better maintenance of quality of life. The assessment of tumor tissue for the expression of programmed death ligand-1 (PD-L1), human epidermal growth factor receptor-2 (HER2) and DNA mismatch repair (MMR) enzymes informs the recommendation for complementing systemic treatment with PD-1-directed immune checkpoint inhibition or HER-2-directed targeted treatment.

## Diagnosis

### Diagnosis

#### Initial diagnosis

Endoscopy is considered the most sensitive and specific diagnostic method. Using high-resolution video-assisted endoscopy, it is possible to detect even discrete changes in color, mucosal surface, and architecture of the gastric mucosa. Endoscopic detection of early lesions can be improved by chromoendoscopy.

The aims of further diagnostics are to determine the stage of the disease and to guide therapy, see Table 1.

**Table 1:** Diagnostic procedures and staging in gastric cancer

Investigation	Note
Physical examination	
laboratory (blood)	Blood count, liver and kidney function parameters, coagulation, tumor markers (CEA, CA 19–9, CA 72–4)
Endoscopy upper gastrointestinal tract	Optional addition of chromoendoscopy
Endoscopic ultrasound (EUS)^1^	For therapy planning in case of localized disease
Computed tomography of thorax, abdomen, and pelvis with oral and intravenous contrast media	For visualization of locoregional and distant tumor spread
Abdominal ultrasound	Complementary to computed tomography
Laparoscopy, if indicated plus cytology^2^	In cT2/cT3/cT4 without evidence of other distant metastases, to detect/exclude peritoneal metastasis

^1^see Chapter 1.1.3.1

^2^Laparoscopy with cytologic examination of the lavage samples helps to detect clinically occult metastasis to the peritoneum in locally resectable tumors. The detection of macroscopic peritoneal metastasis has immediate implications for treatment planning [87]. Cytologic evidence of malignant cells in the lavage samples is an unfavorable prognostic factor, but—outside of clinical studies—has no definite impact on treatment recommendation to date. Laparoscopically abnormal findings are more frequently found in T3/T4 classified tumors [88]

#### Histology and subtypes

Histologic diagnosis of gastric cancer should be made from a biopsy, which is evaluated by two experienced pathologists [1].

##### Laurén classification

Histologically, gastric cancer is characterized by a strong heterogeneity, as several different histological features may be present in one tumor. Over the past decades, histologic classification has been based on the Laurén classification [2]:

- Intestinal type, approximately 54%
- Diffuse type, approx. 32
- Indeterminant, approx. 15%

The diffuse subtype is found more in women and people of younger age, while the intestinal type is more common in men and people of older age and is associated with intestinal metaplasia and *Helicobacter pylori* infection [3].

##### World Health Organization (WHO) classification of gastric cancer

The World Health Organization (WHO) classification distinguishes four definitive types of gastric cancer [4].

- Tubular
- Papillary
- Mucinous
- Poorly cohesive (including signet ring cell carcinoma).

The classification is based on the predominant histologic pattern of the carcinoma, which often coexists with less dominant features or other histologic patterns.

##### The Cancer Genome Atlas (TCGA) classification

Molecular genetic studies divide gastric cancer into molecular subtypes based on studies of the genome, transcriptome, epigenome, and proteome. The most popular molecular subtyping according to TCGA distinguishes four subtypes [5]:

- Chromosomal instability—CIN
- Epstein–Barr virus-associated—EBV
- Microsatellite instability—MSI
- Genomically stable—GS

This classification currently has limited impact on treatment selection.

#### Stages and staging

##### TNM staging

The classification of the extent of the primary tumor and metastasis is based on the UICC/AJCC TNM criteria [2, 4, 6]. Since January 1, 2017, the 8th edition has been used in Europe [4]. The TNM criteria are summarized in Table 2, and the staging is summarized in Table 3.

**Table 2:** UICC-TNM classification of gastric cancer [4]

Classification	Tumor
T	Primary tumor
T1	Superficial infiltrating tumor
T1a	Tumor infiltrating lamina propria or muscularis mucosae
T1b	Tumor infiltrating submucosa
T2	Tumor infiltrating muscularis propria
T3	Tumor infiltrating subserosa without invasion of visceral peritoneum
T4a	Tumor penetrating subserosa (visceral peritoneum)
T4b	Tumor infiltrating adjacent structures
N	Regional lymph nodes
N0	No regional lymph node metastases
N1	Metastases in 1–2 lymph nodes
N2	Metastases in 3–6 lymph nodes
N3a	Metastases in 7–15 lymph nodes
N3b	Metastases in 16 or more lymph nodes
M	Distant metastases
M0	No distant metastases
M1	Distant metastases or positive peritoneal cytology

**Table 3:** Classification of tumor stages [4]

UICC stage	Primary tumor	Lymph nodes	Distant metastases
0	Tis	N0	M0
IA	T1a T1b	N0 N0	M0 M0
IB	T2 T1	N0 N1	M0 M0
IIA	T3 T2 T1	N0 N1 N2	M0 M0 M0
IIB	T4a T3 T2 T1	N0 N1 N2 N3	M0 M0 M0 M0
IIIA	T4a T3 T2	N1 N2 N3	M0 M0 M0
IIIB	T4b T4a T3	N0/1 N2 N3	M0 M0 M0
IIIC	T4b T4a	N2/3 N3	M0 M0
IV	Any T	Any N	M1

Endoscopic ultrasound (EUS) is particularly suitable for determining the clinical T category, as it can best visualize the different layers of the gastric wall. EUS should, therefore, be part of primary staging in a patient with a curative therapeutic approach.

The following characteristics serve to identify malignant lymph nodes on CT slice imaging [7]:

- Diameter ≥ 6–8 mm (shorter axis) in perigastric lymph nodes
- Round shape
- Central necrosis
- Loss of the fat hilus
- Heterogeneous or enhanced contrast agent uptake

The sensitivity of CT for lymph node staging is variably estimated at 62.5–91.9% in systematic reviews [8].

EUS improves the accurate determination of the T and N categories and can help determine the proximal and distal margins of the tumor. EUS is less accurate for tumors of the antrum. EUS is considered more accurate than CT in diagnosing malignant lymph nodes.

Signs of malignancy on EUS include [9]:

- Hypoechoic
- Round shape
- Blurred demarcation from the surrounding area
- Size in the longest diameter > 1 cm

## Therapy

### Therapy structure

Multidisciplinary planning is required for any initial treatment recommendation. It should be developed in a qualified multidisciplinary tumor board.

Core members of the multidisciplinary board include the following disciplines: Visceral Surgery, Medical Oncology, Radiation Oncology, Gastroenterology, Radiology and Pathology. Whenever possible, patients should be treated in clinical trials.

**Fig. 1:**
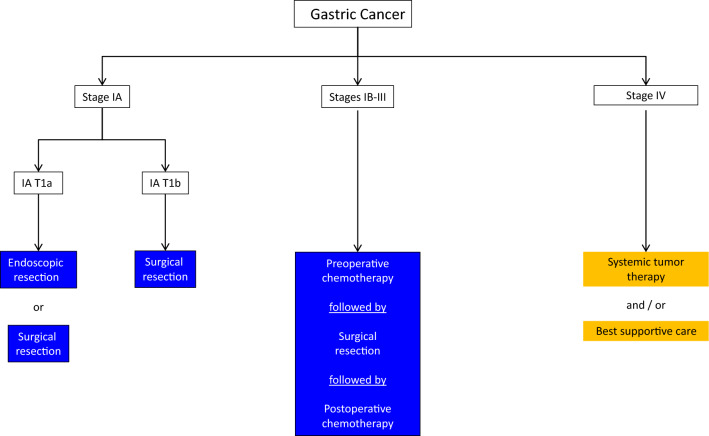
Algorithm for stage-adapted management of gastric cancer

Therapy is stage adapted. A treatment algorithm for the stage-adapted management of gastric cancer is shown in Fig. 1.

#### Stage IA—T1a

Since the probability of lymph node metastasis in mucosal gastric cancer (T1a) is very low, endoscopic resection (ER) may be sufficient [10]. If histopathologic workup after endoscopic resection reveals that tumor infiltration extends into the submucosa (T1b), surgical resection with systematic lymphadenectomy should be performed, as lymph node metastases may already be present in up to 30% of cases.

Gastric cancers classified as pT1a cN0 cM0 should be treated with endoscopic resection, considering the adapted Japanese criteria [1, 11]. A (limited) surgical approach is an alternative.

Perioperative or adjuvant chemotherapy is not indicated for stage IA (T1a) patients.

#### Stage IA—T1b

For stage IA gastric cancer with infiltration of the submucosa, the risk of lymph node metastases is 25–28%. The 5-year survival rate is 70.8% for all stage IA in the SEER database [12], and the cancer-specific survival rate at 10 years is 93% in the Italian IRGGC analysis. Therapy of choice in stage I (T1b category) is radical surgical resection (subtotal, total, or transhiatal extended gastrectomy). Limited resection can be recommended only in exceptional cases due to the imprecise accuracy of pre-therapeutic staging.

A benefit from perioperative or adjuvant chemotherapy has not been established for stage IA (T1b) patients.

#### Stage IB—III

In stage IB—III, resection should consist of radical resection (subtotal, total, or transhiatal extended gastrectomy) in combination with D2- lymphadenectomy. Subtotal gastrectomy can be performed if safe free tumor margins can be achieved. The previously recommended tumor-free margins of 5 and 8 cm for intestinal and diffuse tumor growth types, respectively, are no longer accepted. The scientific evidence for definitive recommendations is low. A negative oral margin in the intraoperative frozen section is crucial.

Perioperative chemotherapy with a platinum derivative, a fluoropyrimidine, and an anthracycline significantly prolonged overall survival in patients with resectable gastric cancer in the MAGIC trial [13]. In the French FNCLCC/FFCD multicenter study, perioperative chemotherapy with a platinum derivative and a fluoropyrimidine without anthracycline showed a comparable effect size on improving survival [14]. Currently, neither chemotherapy regimen is the first choice.

Treatment according to the FLOT regimen (5-fluorouracil/folinic acid/oxaliplatin/docetaxel) further improved progression-free survival (hazard ratio, HR 0.75) and overall survival (HR 0.77) in patients with stage ≥ cT2 and/or cN + compared with therapy analogous to MAGIC. The relatively higher efficacy of FLOT was shown to be consistent across relevant subgroup analyses such as age, histology, and tumor location. The rate of perioperative complications was comparable [15].

For patients with gastric cancer ≥ stage IB who received resection without prior chemotherapy (e.g., due to misdiagnosed tumor stage prior to surgery), adjuvant chemotherapy may be recommended.

In HER2-positive tumors, a benefit from combining perioperative chemotherapy with a HER2 antibody in the perioperative setting in terms of overall survival has not been proven, and therefore cannot be recommended outside of clinical trials. The AIO-PETRARCA phase 2 study showed a higher histopathologic remission rate when FLOT chemotherapy was combined with trastuzumab + pertuzumab and a trend in favor of better progression-free and overall survival [16]. These data require validation in larger and independent cohorts.

In microsatellite instability (MSI-H) localized gastric carcinoma, the efficacy of perioperative chemotherapy, based on retrospective data analyses [17], has been controversially discussed. However, more recent data from the DANTE trial show that complete and subtotal tumor remissions can be achieved with FLOT chemotherapy even in MSI-H subtype gastric carcinomas [18]. Thus, according to the current status, perioperative chemotherapy with the FLOT regimen remains indicated for MSI-H gastric cancers if tumor response is pursued. The FFCD-NEONIPIGA phase 2 study showed a high histopathologic remission rate after 12 weeks of therapy with nivolumab + ipilimumab without chemotherapy in resectable MSI-H cancers [19]. Data require validation in larger and independent patient cohorts.

After R1 resection, adjuvant radiochemotherapy may be considered.

#### Stage IV

The aim of therapy is usually non-curative. The first priority is systemic drug therapy, supplemented in individual cases by local therapeutic measures. Active symptom control and supportive measures such as nutritional counseling, psychosocial support, and palliative care are an integral part of treatment. The prognosis of patients with locally advanced and irresectable or metastatic (pooled here as "advanced") gastric cancer is unfavorable. Studies evaluating the benefit from chemotherapy have shown a median survival of less than 1 year [20]. However, there is evidence that chemotherapy can prolong the survival of patients with advanced gastric cancer compared to best supportive therapy alone and maintain quality of life longer [21].

##### Systemic tumor therapy

**Fig. 2:**
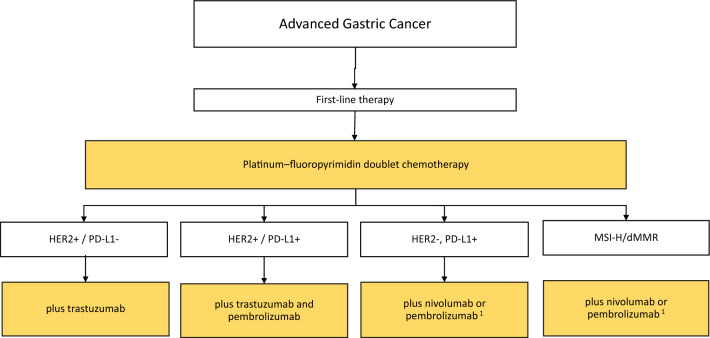
Algorithm for first-line therapy of advanced gastric cancer. ^1^Nivolumab is approved in Europe for PD-L1 CPS ≥ 5 according to Checkmate-649; pembrolizumab is approved in Europe for adenocarcinoma of the esophagus and esophago-gastric junction for PD-L1 CPS ≥ 10 according to Keynote-590. Positive phase III trial results in patients with PD-L1 CPS-positive gastric cancer were also reported from Keynote-859 and subgroup analyses from several first-line studies (Checkmate-649, Keynote-062, Keynote-859) show benefit for nivolumab or pembrolizumab in combination with chemotherapy in patients with MSI-H/dMMR tumors

**Fig. 3:**
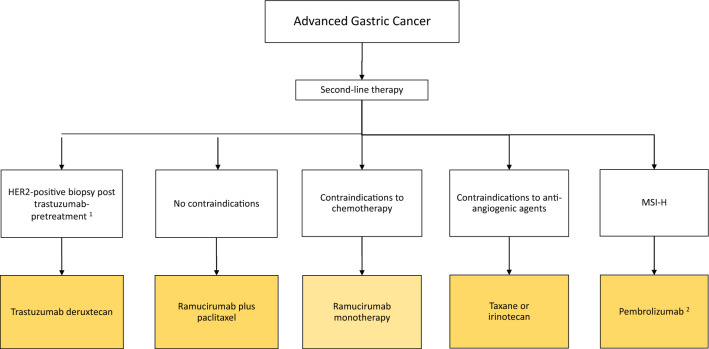
Algorithm for second-line therapy of advanced gastric cancer. ^1^Since many tumors lose HER2 overexpression after trastuzumab failure, reassessment of HER2 status using a fresh biopsy is recommended prior to second-line trastuzumab deruxtecan (T-DXd) therapy. ^2^Pembrolizumab in second line for MSI-high advanced gastric cancer is not recommended when immunotherapy was administered in first-line treatment

**Fig. 4:**
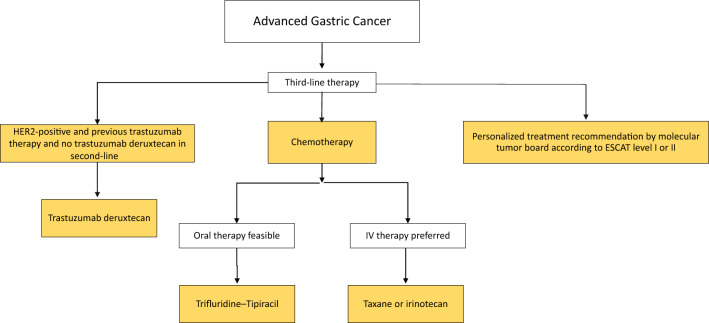
Algorithm for third-line therapy of advanced gastric cancer. ^1^According to the Destiny Gastric 01 study, re-testing of HER2 status is not mandatory for third-line T-DXd therapy, ^2^ if not administered in second-line treatment

The current recommended algorithms for drug therapy of patients with advanced gastric cancer are shown in Figs. 2, 3, and 4.

##### First-line chemotherapy, molecular targeted therapy, and immunotherapy

##### Chemotherapy

The standard of care for first-line chemotherapy of advanced gastric cancer is a platinum–fluoropyrimidine doublet. Oxaliplatin and cisplatin are comparably effective, with a more favorable side effect profile for oxaliplatin. This may contribute to a trend toward better efficacy, especially in patients > 65 years [6, 22]. Fluoropyrimidines can be administered as infusion (5-FU) or orally (capecitabine or S-1). Oral fluoropyrimidines are comparably effective to infused 5-FU [23–26]. Capecitabine is approved in combination with a platinum derivative and has been studied with both cis- and oxaliplatin in European patients. S-1 is established as a standard of care in Japan and approved in Europe for palliative first-line therapy in combination with cisplatin. Infused 5-FU should be preferred over oral medications in patients with dysphagia or other feeding problems. In elderly or frail patients, results of the phase III GO-2 trial support a dose-reduced application of oxaliplatin–fluoropyrimidine chemotherapy (to 80 or 60% of the standard dose from the beginning), resulting in fewer side effects with comparable efficacy [27].

The addition of docetaxel to a platinum–fluoropyrimidine combination (three-weekly DCF regimen) improved radiographic response rates and prolonged overall survival in a historical phase III trial, but also resulted in significantly increased side effects [28]. Other phase II trials examined modified docetaxel–platinum–fluoropyrimidine triplets and showed reduced toxicity compared with DCF in some cases [29–32]. However, the higher response rate of a triplet (37% vs. 25% [28] does not translate into prolonged survival in recent trials, which included effective second-line regimens. In the phase III JCOG1013 trial, patients with advanced gastric cancer received either cisplatin plus S-1 or cisplatin plus S-1 and docetaxel. There were no differences in radiographic response, progression-free survival, or overall survival [33]. Therefore, with increased toxicity and uncertain impact on overall survival, no recommendation can be made for first-line docetaxel–platinum–fluoropyrimidine therapy, so that a platinum–fluoropyrimidine doublet remains the standard approach. In individual cases, e.g., when fast tumor regression is urgently required, first-line therapy with a platinum–fluoropyrimidine–docetaxel triplet may be indicated.

Irinotecan-5-FU has been compared with cisplatin-5-FU and with epirubicin–cisplatin–capecitabine in randomized phase III trials and showed comparable survival with controllable side effects [34, 35]. Irinotecan-5-FU can, therefore, be considered a treatment alternative to platinum–fluoropyrimidine doublets according to scientific evidence; however, irinotecan has no formal approval in Europe for gastric cancer.

##### HER2-positive gastric cancer

HER2 positivity is defined in gastric cancer as the presence of protein expression with immunohistochemistry score [IHC] of 3 + or IHC 2 + and concomitant gene amplification on in situ hybridization [ISH], HER2/CEP17 ratio ≥ 2.0. HER2 diagnosis should be quality controlled [36, 37]. Trastuzumab should be added to chemotherapy in patients with HER2-positive advanced gastric cancer [21, 38]. The recommendation is based on data from the phase III ToGA trial, showing a higher response rate and prolonged survival for trastuzumab–cisplatin–fluoropyrimidine chemotherapy vs. chemotherapy alone using the above selection criteria; the additional trastuzumab side effects are minor and controllable [38]. Combinations of trastuzumab and oxaliplatin plus fluoropyrimidine show comparable results to the historical cisplatin-containing ToGA regimen [39–41]. Based on data from the not yet fully reported results of the Keynote-811 study, the Commission for Human Medical Products (CHMP) of the European Medicines Agency (EMA) published a positive opinion for pembrolizumab plus trastuzumab and chemotherapy as first-line treatment for HER2-positive advanced gastric or gastroesophageal junction (GEJ) adenocarcinoma expressing PD-L1 (CPS ≥ 1) on 20th of July 2023 (https://www.ema.europa.eu/en/medicines/human/summaries-opinion/keytruda-10). If available, this combination should be preferred over trastuzumab plus chemotherapy in the respective patient population (Fig. 2).

##### Immunotherapy

The phase III CheckMate 649 trial evaluated the addition of nivolumab to chemotherapy (capecitabine-oxaliplatin or 5-FU/folinic acid-oxaliplatin) in patients with previously untreated gastric, esophago-gastric junction, or esophageal adenocarcinoma [42]. The study included patients regardless of tumor PD-L1 status; the dual primary endpoints were overall survival and progression-free survival. Approximately 60% of the study population had tumors with a PD-L1 CPS ≥ 5. Nivolumab plus chemotherapy yielded a significant improvement over chemotherapy alone in overall survival (14.4 vs. 11.1 months, HR 0.71 [98.4% CI 0.59–0.86]; *p < *0.0001) and progression-free survival (7.7 vs. 6.0 months, HR 0.68 [98% CI 0.56–0.81]; *p < *0.0001) in patients with a PD-L1 CPS ≥ 5. Overall survival benefit was enriched in patients with MSI-H tumors with nivolumab plus chemotherapy vs. chemotherapy (unstratified hazard ratio 0.38; 95% confidence interval 0.17, 0.84).

The Asian phase II/III ATTRACTION-04 trial also showed a significant improvement in progression-free survival with nivolumab and first-line chemotherapy, but with no significant improvement in overall survival compared to first-line chemotherapy alone. The most likely reason for the lack of survival benefit (> 17 months in both arms) is that many patients received post-progression therapies including immunotherapy after first-line therapy [43].

The multinational randomized phase III Keynote-859 trial included 1589 patients with advanced incurable gastric cancer. Patients received either platinum–fluoropyrimidine plus pembrolizumab or the same chemotherapy plus placebo every 3 weeks. Overall survival was prolonged in the pembrolizumab group (HR 0.78 [95% CI 0.70–0.87], *p < *0.0001). The effect was more pronounced in the subgroup with a PD-L1 CPS ≥ 10 (HR 0.64), whereas efficacy was lower for CPS < 10 (HR 0.86). Overall survival benefit was enriched in patients with MSI-H tumors with pembrolizumab plus chemotherapy vs. chemotherapy (hazard ratio 0.34; 95% confidence interval 0.176, 0.663) [44]. The results, thus, complement the positive trial data from the phase III Keynote-590 study, which led to EU approval of pembrolizumab in combination with platinum–fluoropyrimidine chemotherapy for adenocarcinoma of the esophagus and esophago-gastric junction [45].

Positive phase III trial data were also presented on two immune checkpoint (PD-1) inhibitors not currently approved in Europe. Sintilimab in combination with oxaliplatin and capecitabine improved overall survival in the phase III ORIENT-16 trial [46]. In the phase III Rationale-305 study, tislelizumab prolonged overall survival in combination with platinum–fluoropyrimidine or platinum-investigator-choice chemotherapy in patients with a positive PD-L1 score. PD-L1 was evaluated according to a scoring system not yet established internationally (the so-called Tumor Area Proportion score, TAP) [47]. ORIENT-16 and Rationale-305 have not been fully published to date, but support the overall assessment that PD-1 immune checkpoint inhibitors can improve the efficacy of chemotherapy (depending on PD-L1 expression).

##### Claudin 18.2

Data from the multinational phase III Spotlight trial were recently published. These show that in patients with advanced irresectable gastric cancer and tumor claudin 18.2 expression in ≥ 75% of tumor cells, zolbetuximab, a chimeric monoclonal IgG1 antibody directed against claudin 18.2, in combination with FOLFOX chemotherapy prolongs overall survival (median 18.23 vs. 15.54 months, HR 0.750, *p = *0.0053). The main side effects of zolbetuximab are nausea and vomiting, especially during the first applications [48]. The results of the phase III Spotlight trial are largely confirmed by the multinational phase III GLOW trial, in which the chemotherapy doublet was used as a control therapy or combination partner for zolbetuximab [49]. It remains to be seen whether the European Medicines Agency will grant approval to zolbetuximab in patients with claudin 18.2-positive metastatic and previously untreated gastric cancer.

##### Second-line and third-line therapy chemotherapy and anti-angiogenic therapy

Figures 3 and 4 show the algorithm for second- and third-line therapy for patients with advanced gastric cancer. The evidence-based chemotherapy options in this setting are paclitaxel, docetaxel, and irinotecan, which have comparable efficacy with different specific toxicities [21, 50–52]. Irinotecan may be preferred in patients with preexisting neuropathy; however, there is no EU approval. 5-FU/folinic acid plus irinotecan (FOLFIRI) is also occasionally used, but the scientific evidence for its use in second- and third-line treatment is limited [53]. Ramucirumab plus paclitaxel is the recommended standard for second-line therapy and is approved in the EU. The addition of the anti-vascular endothelial growth factor receptor-2 (VEGFR-2) antibody ramucirumab to paclitaxel increases tumor response rates and prolongs progression-free and overall survival according to the results of the phase III RAINBOW trial [54]. Already in the phase III REGARD trial, ramucirumab monotherapy showed prolonged survival compared to placebo, albeit with a low radiological response rate [55].

##### Immunotherapy in second- and third-line therapy

In the phase III KEYNOTE-061 trial, pembrolizumab monotherapy did not show prolonged overall survival compared with chemotherapy [56]. However, an exploratory subgroup analysis recognized a clear benefit for anti-PD-1 immunotherapy in patients with MSI-H gastric cancer [57]. Therefore, PD-1 inhibition is recommended in advanced MSI-H carcinomas at the latest in second-line treatment. Pembrolizumab has European approval for this indication based on the Keynote-061 and Keynote-158 trials [58]. Of note, pembrolizumab in second line for MSI-High advanced gastric cancer is not recommended when immunotherapy was administered in first-line treatment. Other biomarkers, particularly EBV and tumor mutation burden, are also discussed as predictive factors for PD-1 immune checkpoint inhibitor efficacy [59–61]. However, the evidence to date is insufficient to support a positive recommendation for immunotherapy based upon the presence of these biomarkers.

##### HER2-targeted therapy

Studies evaluating trastuzumab, lapatinib, and trastuzumab emtansine for second-line treatment in patients with HER2-positive carcinomas were negative [62–65]. Therefore, these drugs should not be used in gastric cancer outside of clinical trials. A randomized phase II trial showed an improvement in tumor response rate and overall survival for the antibody–drug conjugate trastuzumab deruxtecan (T-DXd) compared with standard chemotherapy in patients with pretreated HER2-positive advanced gastric cancer [66]. Destiny-GC-04 is an ongoing study, assessing the efficacy and safety of T-DXd compared with ramucirumab and paclitaxel in participants with HER2-positive (defined as immunohistochemistry [IHC] 3 + or IHC 2 + /in situ hybridization [ISH] +) gastric or esophago-gastric junction adenocarcinoma who have progressed on or after a trastuzumab-containing regimen and have not received any additional systemic therapy (https://classic.clinicaltrials.gov/ct2/show/NCT04704934).

Prerequisites for inclusion in the Destiny-GC-01 study were at least two prior lines of therapy, prior treatment with a platinum derivative, a fluoropyrimidine, and trastuzumab, and previously confirmed HER2 positivity. The study was recruited exclusively in East Asia. The results of Destiny-GC-01 were largely confirmed in the single-arm phase II Destiny-GC-02 trial, which included non-Asian patients in second-line therapy. Mandatory was platinum–fluoropyrimidine–trastuzumab pretreatment and confirmed HER2 positivity of the tumor in a recent re-biopsy before initiating T-DXd therapy [67].

The EU approval includes the following indication of T-DXd: monotherapy for the treatment of adult patients with advanced HER2-positive adenocarcinoma of the stomach or esophago-gastric junction who have received a prior trastuzumab-based regimen.

We recommend, according to the classically established HER2 diagnostic criteria, to check the HER2 status prior to therapy with T-DXd, especially if use in second-line therapy is planned, where a valid alternative with paclitaxel–ramucirumab is available. This recommendation is based on the inclusion criteria of the Destiny-GC-02 trial and the knowledge that loss of HER2 status occurs in approximately 30% of gastric cancers after first-line therapy with trastuzumab [62].

There is initial evidence of efficacy of T-DXd in low HER2 expression [68]. However, data are not yet sufficient to recommend its use.

##### Third-line therapy

For the treatment of patients with advanced gastric cancer in the third line and beyond, the best evidence is available for trifluridine–tipiracil (FTD/TPI) based on the phase III TAGS trial. Median overall survival with FTD/TPI vs. placebo was significantly improved in the overall patient cohort, in the third-line cohort, and in the fourth-line cohort [69–71]. Therefore, if oral therapy is feasible, trifluridine–tipiracil (FTD/TPI) should be used; alternatively, if intravenous therapy is preferred, irinotecan or a taxane can be given, if not already used in a previous line of therapy. As shown above, T-DXd is a very effective third-line therapy for HER2-positive carcinoma after trastuzumab pretreatment. Nivolumab also proved to be effective; however, the data from the ATTRACTION-02 trial were obtained exclusively in Asian patients [72], so that nivolumab in the third line of treatment in patients with advanced gastric cancer does not have EMA approval, and therefore cannot be recommended.

Following the recommendation of a molecular tumor board, an unapproved therapeutic option may also be preferred in justified cases, especially if the recommendation can be based on an ESMO Scale for Clinical Actionability of Molecular Targets (ESCAT) level I or II [73].

##### Surgery for metastatic gastric cancer

The randomized phase III REGATTA trial showed that gastrectomy in addition to chemotherapy for metastatic disease did not confer a survival benefit compared with chemotherapy alone [74]. International data analyses show that surgical therapy for oligometastasic disease is increasingly perceived as a treatment option [75–77]. The AIO-FLOT3 phase II trial reported results on the feasibility of resection for stage IV gastric cancer and survival in highly selected patients with oligometastatic disease that was without primary progression on FLOT chemotherapy [78]. The potential prognostic benefit of resections for oligometastatic gastric cancer is currently being evaluated in randomized phase III trials [RENAISSANCE (NCT0257836) and SURGIGAST (NCT03042169)].

In a Delphi procedure, a definition for oligometastasis was determined in a European expert group (OMEC). According to this definition, oligometastasis can be defined as the following phenotypes: 1–2 metastases in either liver, lung, retroperitoneal lymph nodes, adrenal glands, soft tissue or bone [77].

##### Supportive therapy and nutrition

It is recommended that nutritional and symptom screening with appropriate tools be performed regularly in all patients with advanced gastric cancer, and appropriate supportive therapies be derived. A study from China showed that early integration of supportive-palliative care is effective and suggests a survival benefit in patients with advanced gastric cancer [79].

Weight loss is a multifactorial phenomenon and may be due to digestive tract obstruction, malabsorption, or hypermetabolism. Clinical data sets show that weight loss of ≥ 10% before chemotherapy or ≥ 3% during the first cycle of chemotherapy is associated with poorer survival [80]. Also, a change in body composition with impaired muscular capacity was shown to be prognostically unfavorable in patients with advanced gastric cancer [81]. The modified Glasgow Prognostic Score (serum CRP and albumin) can be used to assess the extent of sarcopenia and the prognosis of patients with advanced gastric cancer [82].

From this, it can be concluded that screening for nutritional status should be performed in all patients with advanced gastric cancer (for example, using Nutritional Risk Screening, NRS) [83] and expert nutritional counseling and co-supervision should be offered, if nutritional deficiency is evident.

Dysphagia in proximal gastric cancer can be improved with radiotherapy or stent insertion [84]. Single-dose brachytherapy is the preferred option at some centers and results in longer-lasting symptom control and fewer complications than stent insertion. Stenting is needed for severe dysphagia and especially in patients with limited life expectancy, as the effects of the stent are immediate, whereas radiotherapy improves dysphagic symptoms only after approximately 4–6 weeks [85]. If radiotherapy or a stent are not an option, enteral nutrition via naso-gastric, naso-jejunal, or percutaneously placed feeding tubes may provide relief [86]. The indication for parenteral nutrition follows generally accepted guidelines.
